# A child with hypertension and ambiguous genitalia – an uncommon variant of congenital adrenal hyperplasia: a case report

**DOI:** 10.1186/s13256-017-1341-0

**Published:** 2017-06-23

**Authors:** Vivek Pant, Suman Baral, Bishal Shrestha, Arjun Tumbapo

**Affiliations:** 1 Department of Biochemistry, Institute of Medicine (IOM), Kathmandu, Nepal; 2 Unit of Endocrinology, Department of Medicine, IOM, Kathmandu, Nepal; 3 Department of Medicine, IOM, Kathmandu, Nepal

**Keywords:** 11-beta-hydroxylase deficiency, Congenital adrenal hyperplasia, Hypertension, Puberty

## Abstract

**Background:**

Deficiency in 11β-hydroxylase as a cause of congenital adrenal hyperplasia is uncommon. It should be considered in the differential diagnosis of hypertension with virilization in any prepubescent child.

**Case presentation:**

A 12-year-old Asian boy from eastern Nepal presented with pain in his abdomen and hypertension. He was raised as a male but had absent testicles since birth and had precocious puberty. Plasma testosterone, follicle-stimulating hormone, and luteinizing hormone were below baseline level. Basal 17-hydroxyprogesterone was elevated. Magnetic resonance imaging of his pelvis showed presence of Müllerian structures and karyotyping revealed 46,XX genotype. A clinical diagnosis of 11β-hydroxylase deficiency was made in view of hypertension with severe virilization in a 46,XX individual. Our patient’s legal guardian was unwilling for our patient to change gender and because our patient is underage, the condition was well explained to his parents. He was managed with steroids and antihypertensive drugs. He was on regular follow-up; after 2 years there was no hypertension but he developed true puberty with functional ovaries. He was prescribed leuprolide (gonadotropin-releasing hormone analogue), letrozole (aromatase inhibitor), and a continuation of antihypertensive drugs.

**Conclusions:**

This case highlights the importance of a thorough physical examination of the external genitalia at birth and appropriate referral, and addresses issues in the management of such a disorder. Ethical issues pertaining to consent and who is entitled to give it should be clear so that the affected individual will have optimal psychological development and quality of life.

## Background

Congenital adrenal hyperplasia (CAH) consists of a group of disorders of adrenal steroidogenesis. CAH due to 11β-hydroxylase deficiency is an autosomal recessively inherited disorder, characterized biochemically by increased concentrations of deoxycorticosterone, 11-deoxycortisol, and delta-4-androstenedione and decreased plasma renin concentration. On clinical examination it is associated with hypertension and genital ambiguity in affected females [1]. Approximately two-thirds of patients with 11β-hydroxylase deficiency become hypertensive and this may take several years [2]. Hypertension is the single clinical feature distinguishing 11β-hydroxylase deficiency from 21-hydroxylase deficiency which is the commonest form of CAH, accounting for 90% of cases [3]. Although 11β-hydroxylase deficient CAH is uncommon, it should be considered in the differential diagnosis of hypertension with virilization in any prepubescent child. The purpose of this paper is to increase the awareness of health personnel about CAH, to highlight the importance of early diagnosis and treatment to avoid gender identity disorder that will prevail later, and to discuss the difficult ethical and management aspects.

## Case presentation

### Clinical presentation

An Asian boy from eastern Nepal, who is now 12-years old, was delivered in a local hospital in Jhapa (Eastern region in Nepal, Asia) and was discharged after 1 week of hospitalization for neonatal jaundice which appeared on the second day of life. His mother queried the baby’s apparently smaller phallus, the opening of urethra on undersurface of glans penis, and empty scrotum. The health care providers in the hospital reassured her that it was a normal variant and the testis will descend with time. However, his phallus did not increase in size as expected and his scrotum was still empty. This forced the parents to consult again when he was at the age of 24 months. They were again reassured. They did not know where to go for further advice and hoped that development would be normal with time. Then he did not have developmental milestones like other children. He began to walk at age of 2 years and spoke only words of one or two syllables. In addition, he had progressive hearing impairment on both sides from the age of 4 years. However, he has normal vision, gait, and intellect with good school performance. His mother noted that he had an increased black complexion all over since the age of 3. By the age of 5 years he was found to be taller than most of his peers and around the same time he developed deepening of voice and axillary and facial hair appeared. There is no history of blood transfusion or any prolonged medication. There is no history of maternal ingestion of androgens during pregnancy. He has one elder brother who is normal and healthy. There was no maternal history of hirsutism, genital abnormality, or menstrual irregularity. The family history is negative for infertility, ambiguous genitalia, or unexplained neonatal death.

He is now 12-years old and is attending school. He prefers to wear a t-shirt and jeans; he feels more comfortable playing with boys, watching wrestling, and playing football.

At the age of 8 years he developed abdominal pain. He was referred to a tertiary center for evaluation of his abdominal pain. On presentation, his weight was 39 kg, height was 136 cm with upper segment to lower segment ratio of 75:61 cm. His body mass index (BMI) was 21.08 (which falls between 3rd and 97th percentile). Pallor was present. His blood pressure (BP) was 150/100 mmHg with grade two hypertensive retinopathies. There was a masculine look with mustache; no hypertelorism was present.

**Fig. 1:**
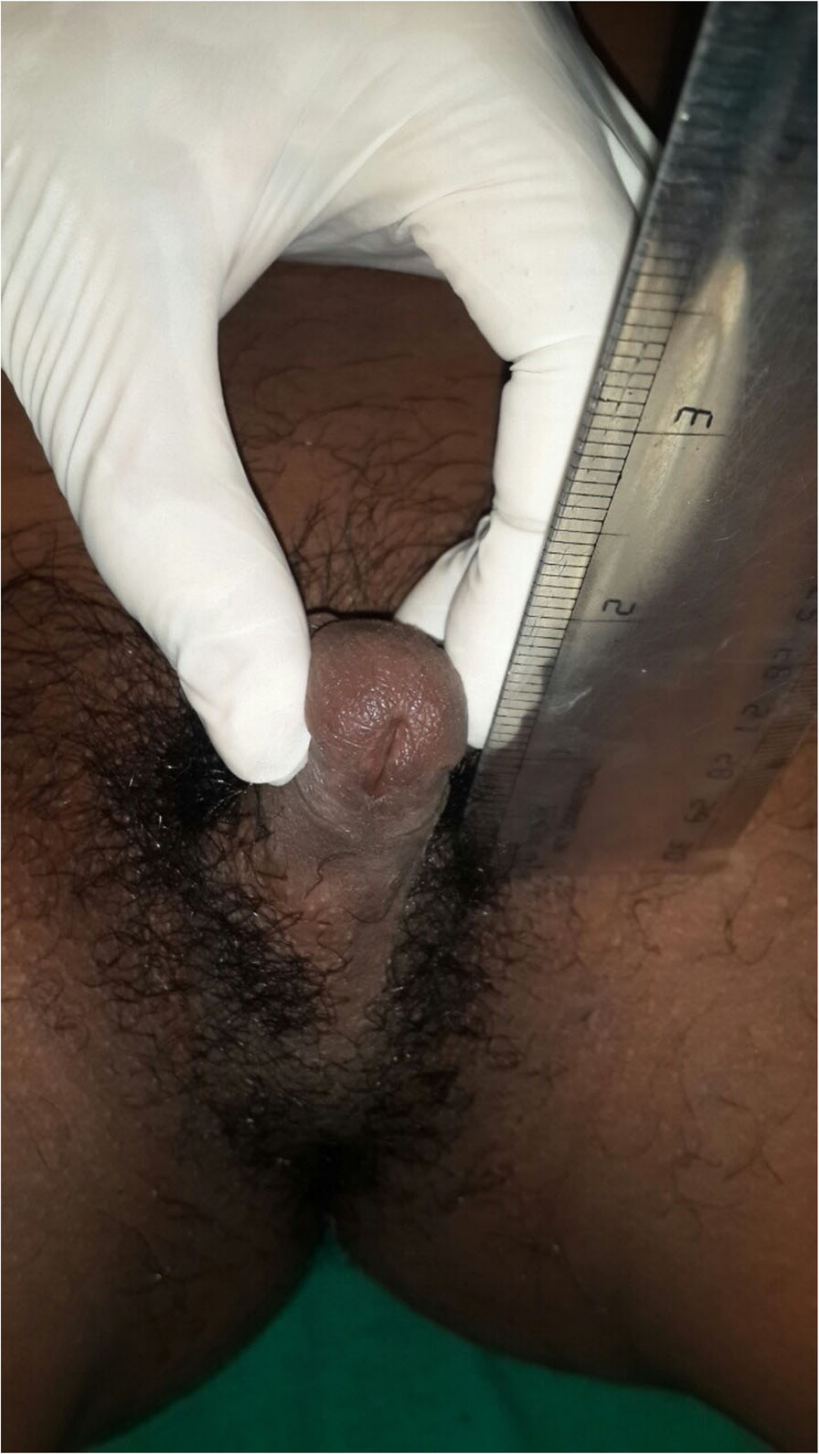
Ambiguous genitalia with Prader score IV

On genital examination, the child had ambiguous genitalia with 3-cm long protrusion and presence of hypospadias with Prader score IV. No testis was palpable in his scrotum, inguinal canal, thigh, or perineum and his anus was normal in position. Tanner stage IV male pattern pubic hair was seen (Fig. 1).

**Fig. 2:**
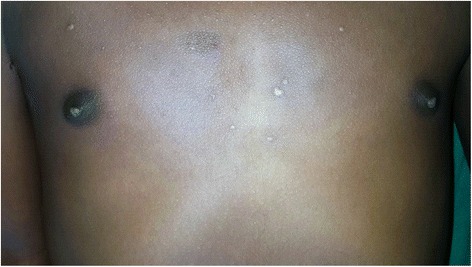
No gynecomastia at age of 8 years

There was no gynecomastia (Fig. 2).

### Investigations

Laboratory investigations revealed normal serum urea and creatinine and normal serum electrolytes. He had low serum cortisol of 1.1 μg/dL at 8 a.m. (reference 4.46 to 22.7 μg/dL) but increased 17-hydroxyprogesterone of 4.13 ng/ml (reference 0.07 to 1.70) and adrenocorticotropic hormone (ACTH) level of 691.0 pg/ml (reference ≤46.0 pg/ml). A test for 11-deoxycorticosterone was not done due to financial constraints.

There was mild concentric left ventricular hypertrophy on echocardiography suggesting hypertensive heart disease. There was presence of hemolytic anemia with iron deficiency, hemoglobin E (HbE) variant of thalassemia. There was also severe (76 db) sensorineural hearing loss of the right ear and profound (83 db) sensorineural hearing loss of the left ear.

**Fig. 3:**
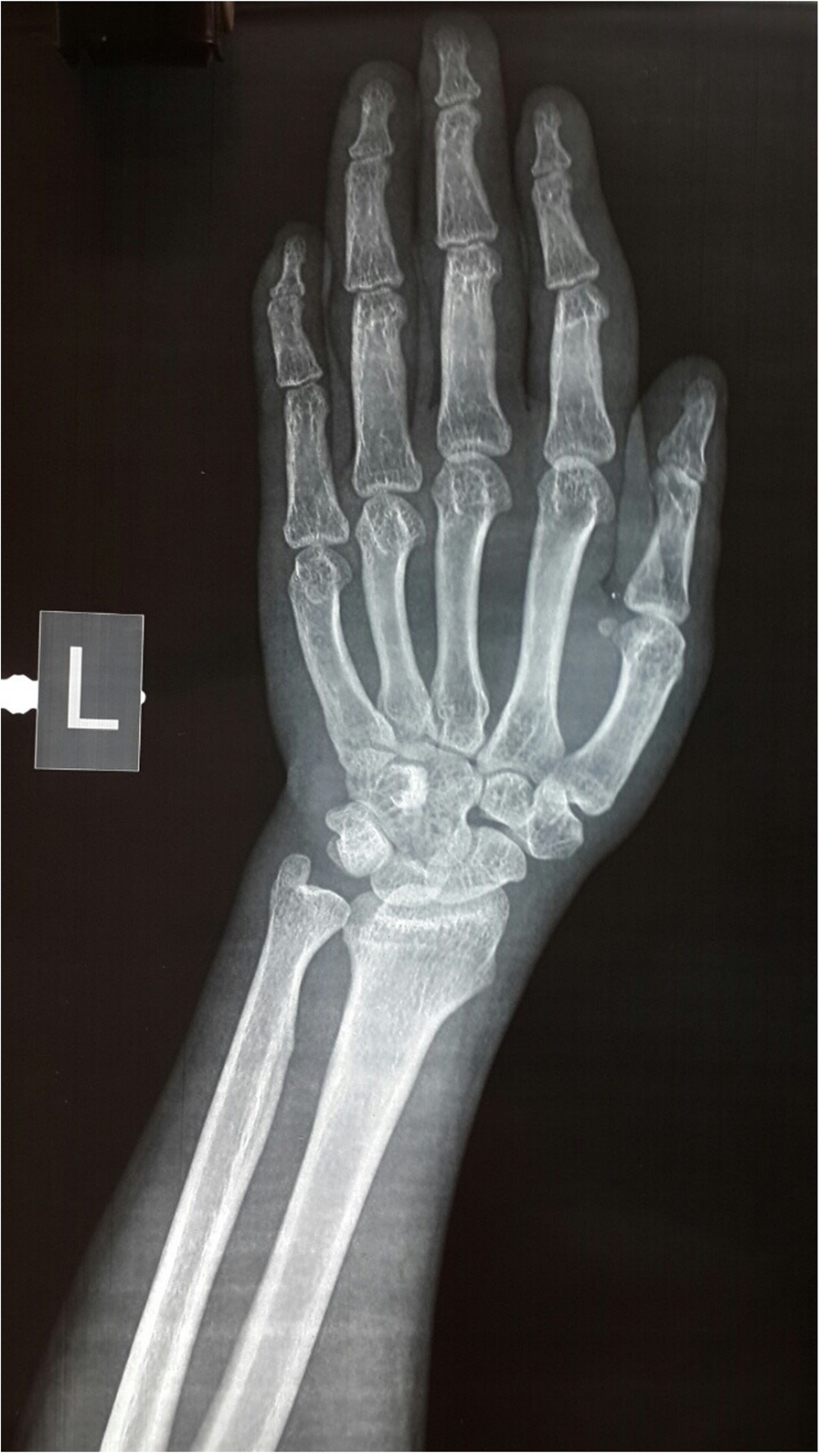
X-ray of left hand showing epiphyseal fusion. The lower end of the radius shows epiphyseal closure and ulna also shows epiphyseal closure (bone age is intermediate between 17 and 18)

An X-ray of his left hand revealed premature closure of epiphysis (Fig. 3).

Ultrasonography of his abdomen/pelvis revealed a uterus and there was significant doubt about whether an inguinal mass was a testis or lymph node. Solitary cholelithiasis was also noted.

**Fig. 4:**
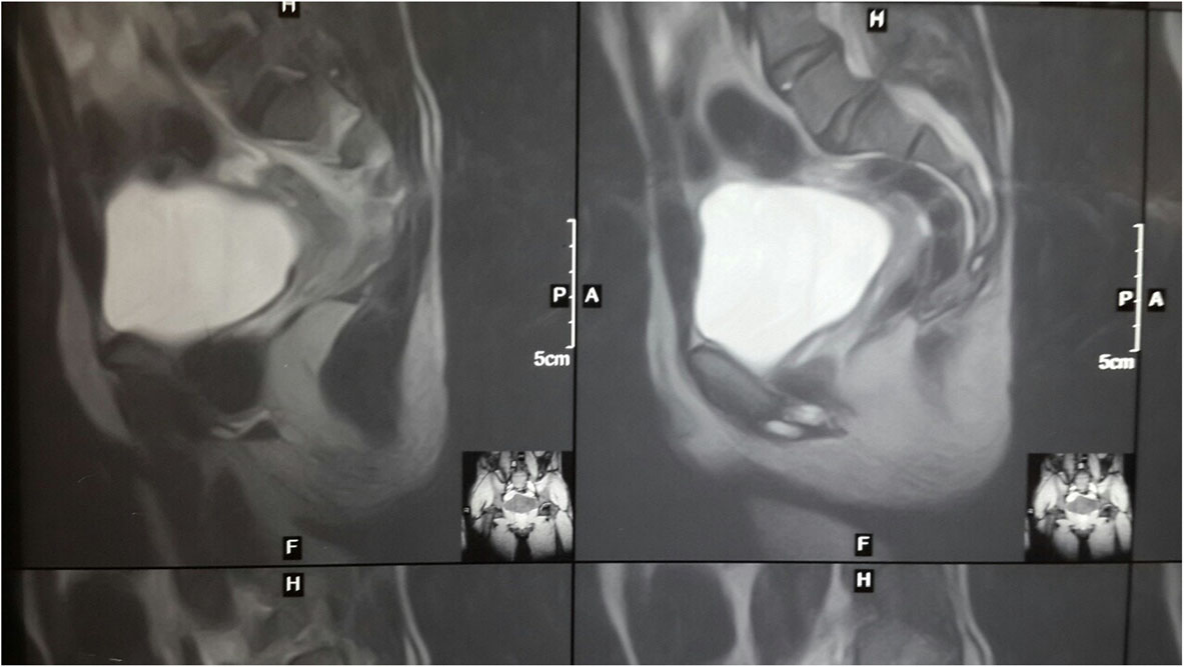
Magnetic resonance imaging of the pelvis showing Müllerian structures

Magnetic resonance imaging of his abdomen/pelvis revealed presence of Müllerian structures and no testis was seen (Fig. 4). Karyotyping revealed 46,XX chromosome.

Thus, he was diagnosed as having CAH with 11β-hydroxylase deficiency, ambiguous genitalia, hypertension with hypertensive heart disease, sensorineural hearing loss, and hemoglobinopathy (β thalassemia HbE variant).

### Management

There was a treatment dilemma as anti-androgenic medicine might reveal female characteristics. The patient's parents were counselled about the disadvantages of sex change to genotypic sex, fertility issues, sexual functioning, and expensive surgeries. Possible features of gender dysphoria and phenotyping changes on not initiating treatment were also explained. Our patient’s legal guardian was unwilling for our patient to change gender and the patient himself is underage. As our patient is a minor, the condition was well explained to his parents. Our patient was put on cortisone tablet 5 mg at bedtime and spironolactone tablet 25 mg along with amlodipine 5 mg. He responded well to the treatment and his BP came down to 100/60 mmHg; he has been in follow-up for the past 2 years and presently is not having any side effects.

**Fig. 5:**
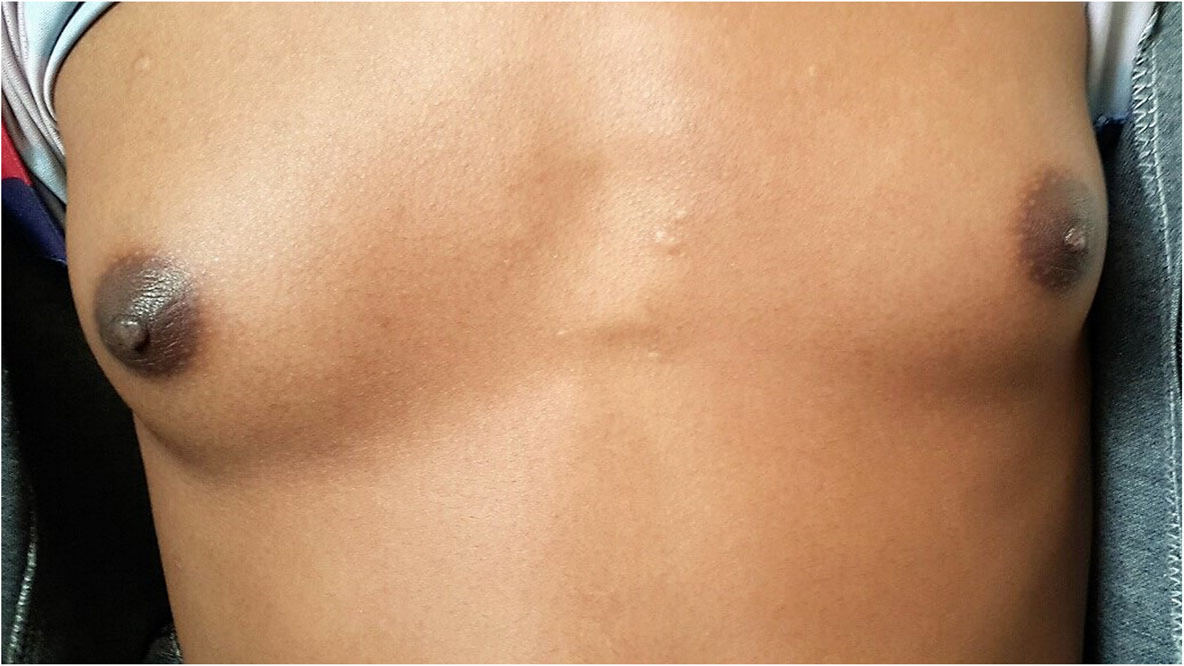
Thelarche

He is now 12-years old. On examination thelarche was seen (Fig. 5). His serum luteinizing hormone (LH) and follicle-stimulating hormone (FSH) have increased. His serum cortisol is still in lower range. So, he was prescribed with leuprolide, which is a gonadotropin-releasing hormone (GnRH) analogue, to decrease LH and FSH, letrozole (aromatase inhibitor) to decrease his estrogen level, and the dose of spironolactone was adjusted. His serum iron and ferritin were within normal range.

## Discussion

Deficiency in 11β-hydroxylase is the second most common cause of CAH [4]. Mutation of *CYP11B1* gene causes decreased activity or inactivation of enzyme, which causes a decrease in conversion of 11-deoxycorticosterone to corticosterone and 11-deoxycortisol to cortisol [5]. Reduced cortisol now increases ACTH secretion through feedback mechanism. There is overproduction of precursors proximal to the enzyme defect which serve as substrates for the accelerated androgen pathways, so that adrenal androgen secretion is increased [2]. In such a case, development of the female external genitalia is affected *in utero* by excess fetal adrenal androgens, resulting in genitalia which are ambiguous. Females are so severely virilized at birth that their external genitalia are male looking, with a penile urethra and fused labioscrotal folds [2]. This leads to error in gender assignment after birth as in our case. Due to a delay in diagnosis in our patient, our patient was raised as a boy. Elevated metabolites with mineralocorticoid activity, such as deoxycorticosterone and its derivatives, cause hypertension in approximately two-thirds of patients [2]. In our case, our patient has hypertension and development of hypertensive heart disease at diagnosis. Patients undergo rapid somatic growth with premature epiphyseal closure. Pelvic imaging revealed presence of Müllerian structures in our case. Unlike the external genitalia, gonads and internal structures (ovarian tubes, uterus, and cervix) that are derivatives of the Müllerian ducts are preserved since the substance that normally causes involution of these structures in men (Müllerian inhibiting factor) is not produced by the fetal ovary [6]. Biochemical findings and an imaging report along with clinical features led to the diagnosis of 11β-hydroxylase deficiency. Due to financial constraints, we were unable to perform plasma levels of 11-deoxycortisol and renin which would have conclusively proven the diagnosis, but, as described earlier, clinical and available biochemical results supported the diagnosis of CAH due to 11β-hydroxylase deficiency. CAH caused by 11β-hydroxylase deficiency has several mutations that may affect heme binding sites. Mutations that altered the heme binding site, such as R374W and R448H/C, resulted in high Prader scores (4/5), severe hypertension, and profoundly advanced bone age [7]. These all are present in our case.

If the diagnosis is late, it is a dilemma whether to change the gender. The decision depends on the age at diagnosis. To preserve fertility, female assignment is recommended by most authorities in all 46,XX cases of CAH [8]. But in our case, the time at which the initial diagnosis was suspected was too late for his parents to accept a new gender. Further, the ethical aspect, regarding performance of elective genital surgeries is challenging. Elective genital surgeries should not be considered until the affected individual is old enough to possess the intellectual capacity to decide [9]. Also, early castration requires post-pubertal hormone replacement therapy, which poses its own risks such as elevated rates of cardiovascular disease, cancers, or osteoporosis [10]. In cases in which the patient is a minor, as in our case, every aspect such as the disadvantage of sex change to genotypic sex, fertility issues, sexual functioning, expensive surgeries, and features of gender dysphoria should be clearly explained to the legal guardians. There is consensus to rear child as male when the individual has XX genotype with CAH with extensively fused labia and a penile clitoris [11]. The legal guardian of our patient decided to retain the male gender identity of our patient, which was assigned at birth. Medical management for us is challenging since the child has now developed true puberty. The World Professional Association for Transgender Health (WPATH) that publishes a Standards of Care (SOC) document states that adolescents applying for hormone treatment and surgery should satisfy two sets of criteria which are eligibility and readiness before proceeding [12]. In our case, the child’s serum estrogen, LH, and FSH have started rising signifying that ovaries have started to function. So, the addition of GnRH analogues was considered. In general, transsexual adolescents (Tanner stage 2) are treated by suppressing puberty with GnRH analogues until they are 16-years old, after which cross-sex hormones may be given [13]. So, our patient was prescribed leuprolide (GnRH analogue) to decrease LH and FSH, letrozole (aromatase inhibitor) to decrease estrogen level, and his dose of spironolactone was adjusted. The challenges we faced in the management of this patient were ethical issues pertaining to consent, expensive drugs, and timely follow-up.

## Conclusions

The gender that is assigned to a child at birth should not be based on mere inspection of the external genitalia since they may be ambiguous as well as incongruent with the internal anatomy. Protocols regarding medical management of such cases, together with ethical issues pertaining to consent and who is entitled to give it, should be clear to facilitate optimal psychological development and quality of life for the affected individual.
